# Membrane Transport: Ionic Environments, Signal Transduction, and Development of Therapeutic Targets

**DOI:** 10.1155/2015/581626

**Published:** 2015-04-12

**Authors:** Akio Tomoda, Yoshinori Marunaka, Douglas C. Eaton, Anuwat Dinudom

**Affiliations:** ^1^Tokyo Medical University, Tokyo 160-0022, Japan; ^2^Departments of Molecular Cell Physiology and Bio-Ionomics, Kyoto Prefectural University of Medicine, Kyoto 602-8566, Japan; ^3^Japan Institute for Food Education and Health, St. Agnes' University, Kyoto 602-8013, Japan; ^4^Department of Physiology, Emory University School of Medicine, Atlanta, GA 30322, USA; ^5^Department of Physiology, Sydney Medical School, University of Sydney, Sydney, NSW 2006, Australia

Membrane transport plays a critical role in producing ionic environments in both intracellular and extracellular spaces that are necessary for cellular signal transduction. The importance of membrane transport implies that any abnormality may lead to pathophysiological conditions and often to specific diseases. This also means that membrane transporters, including ion channels and transporters, are very important therapeutic targets in several diseases such as cardiovascular disorders, hypertension, dementia, metabolic syndrome associated with diabetes mellitus, and cancer. Thus, many researchers have been investigating regulatory mechanisms of membrane transport with an aim to develop new therapies for treating transport disorders found in various diseases.

A specific example of a transport defect associated with serious pathology involves abnormalities in total body sodium balance. Na^+^ that is transported via the epithelial Na^+^ channel (ENaC) [1–3] determines the body fluid volume, blood pressure, and the amount of fluid in the alveolar space. The positive potential gradient generated by transepithelial Na^+^ movement (reabsorption) drives movements of anions. The osmotic gradient produced by movement of salt promotes water reabsorption [4]. Patients with Liddle's syndrome have a gain of function mutation in ENaC leading to hypertension due to excess body fluid volume, caused by abnormally high ENaC-mediated Na^+^ reabsorption [5], showing that the activity of ENaC is involved in the regulation of blood pressure. ENaC-mediated Na^+^ reabsorption is mainly determined by the number of ENaC expressed at the apical cell membrane, which is regulated by intracellular trafficking of ENaC proteins. In addition, the activity of ENaC is also regulated by protease-mediated cleavage of ENaC subunits [4]. Of course, biosynthesis of ENaC proteins is one of the most important regulatory factors that determine total activity. Several humoral agents including vasopressin, catecholamine, osmolarity, and aldosterone are known to regulate expression of ENaC at the plasma (apical) membrane [4, 6, 7].

Epithelial Cl^−^ transport (secretion) has been known to play an important role in the regulation of fluid volume in the lung [8–10]. Epithelial Cl^−^ transport is a two-step process: (1) the Cl^−^-uptake from the interstitium into the cytosolic space across the basolateral membrane, which is mediated by the Na^+^-K^+^-2Cl^−^ cotransporter (NKCC), and (2) the Cl^−^-releasing step across the apical membrane via apical Cl^−^ channels such as the cystic fibrosis transmembrane conductance regulator (CFTR) Cl^−^ channel and the Ca^2+^-activated Cl^−^ channels. Activities of NKCC and Cl^−^ channels are known to be regulated by various factors, including catecholamines and insulin [10].

Cytosolic Cl^−^ is involved in various cellular functions such as gene expression, neuron elongation, and cancer cell growth [11, 12]. In addition, mRNA expression of ENaC is regulated by cytosolic Cl^−^. Quercetin (a flavonoid) that elevates cytosolic Cl^−^ concentration by activating NKCC has been known to downregulate mRNA expression of ENaC. Furthermore, elongation of neurites depends on the concentration of cytosolic Cl^−^. It has been reported that Cl^−^ enhances tubulin polymerization by inhibiting activity of GTPase contained in tubulin molecule. Moreover, changes in the activity of ion transporters and channels regulate cancer cell growth by modulating the cytosolic Cl^−^ concentration via control of MAPK-mediated signaling pathways.

pH is one of the most important factors that regulate cell function and enzyme activity [13–15]. It is well established that the intracellular pH is finely regulated by various types of H^+^ transporters, such as the Na^+^/H^+^ exchanger. Although the physiological role of extracellular (interstitial) pH has not been studied extensively, extracellular (interstitial) pH has recently been recognized as an essential factor in several pathophysiological conditions and insulin resistance in diabetes mellitus.

Many investigators have studied cell volume regulatory mechanisms. The physiological and pathophysiological significance of cell volume regulation including regulatory cell volume decrease (RVD) have recently been described [16]. Chemosensing process and signal transduction of immune cells are mediated via membrane transport. More detail is available in this issue.

The roles of membrane transporters in the regulation of cellular functions described above has addressed some, but not by any means all, of the significance of the transporters as key factors in maintaining the homeostasis of body compartments and functions. Membrane transporters, including ion channels and transporters, are known targets for treatment of various diseases. We hope that readers of this special issue will find not only new data on membrane transport and its roles and updated reviews on membrane transport mechanisms, but also concepts and new ideas for developing new therapies for various types of diseases.

*Akio Tomoda* *Yoshinori Marunaka* *Douglas C. Eaton* *Anuwat Dinudom*
